# Plant-Based Proteins and Renal Protection in Acute Kidney Injury: Nutritional and Metabolic Perspectives

**DOI:** 10.3390/nu18091395

**Published:** 2026-04-29

**Authors:** Diana Zarantonello, Sergio Lassola, Andrea Carta, Omar Fathalli, Silvia De Rosa

**Affiliations:** 1 Department of Nephrology, Santa Chiara Hospital, Azienda Sanitaria Universitaria Integrata del Trentino (ASUIT), 38122 Trento, Italy; diana.zarantonello@asuit.tn.it (D.Z.); andrea.carta@asuit.tn.it (A.C.); 2 Department of Anesthesia and Intensive Care, Santa Chiara Hospital, Azienda Sanitaria Universitaria Integrata del Trentino (ASUIT), 38122 Trento, Italy; sergio.lassola@asuit.tn.it; 3 Centre for Medical Sciences—CISMed, University of Trento, 38122 Trento, Italy; omar.fathalli@unitn.it

**Keywords:** acute kidney injury, plant-based proteins, renal nutrition, protein-energy wasting, amino acid metabolism, gut–kidney axis, enteral nutrition, nephroprotection

## Abstract

Acute kidney injury (AKI) is a frequent complication in critically ill patients and is associated with high morbidity, mortality, and an increased risk of progression to chronic kidney disease (CKD). In this context, nutritional management represents a key component of supportive therapy, as AKI is commonly characterized by hypercatabolism, negative nitrogen balance, and protein-energy wasting. Current nutritional strategies primarily focus on the quantity of protein intake required to compensate for catabolic losses, particularly in patients undergoing renal replacement therapy (RRT). However, growing evidence suggests that the quality and metabolic effects of dietary protein sources may also influence renal physiology and recovery. Plant-based proteins have recently gained attention as a potentially advantageous nutritional strategy in kidney disease. Compared with animal-derived proteins, plant-based proteins are associated with a lower dietary acid load, reduced production of gut-derived uremic toxins, and beneficial effects on the intestinal microbiota. In addition, their amino acid profile may modulate oxidative stress, inflammatory pathways, and renal hemodynamics. These characteristics may contribute to a more favorable metabolic environment in patients with AKI, potentially supporting renal recovery and reducing the risk of AKI-to-CKD transition. This review examines the pathophysiological mechanisms linking protein metabolism, renal injury, and nutritional support in AKI. Particular attention is given to the role of plant-based proteins, their amino acid composition, and their potential nephroprotective effects. Understanding the interaction between dietary protein sources, metabolic pathways, and the gut–kidney axis may help guide future nutritional strategies aimed at improving outcomes in critically ill patients with AKI.

## 1. Introduction

Acute kidney injury (AKI) is a frequent complication in critically ill patients and is associated with significant morbidity, mortality, prolonged hospitalization, and an increased risk of progression to chronic kidney disease (CKD) [1]. The incidence of AKI in intensive care units (ICUs) ranges from 30% to 60%, depending on patient population and diagnostic criteria, and up to 10–15% of patients require renal replacement therapy (RRT) [2].

Beyond its direct impact on renal function, AKI is increasingly recognized as a systemic condition involving complex metabolic, inflammatory, and hemodynamic alterations that influence multiple organ systems and clinical outcomes [3].

Among these alterations, profound changes in protein metabolism represent a central feature of critical illness and AKI [4]. Critically ill patients often develop a hypercatabolic state driven by systemic inflammation, hormonal stress responses, and mitochondrial dysfunction [5]. This condition leads to accelerated skeletal muscle breakdown, increased amino acid mobilization, and negative nitrogen balance [6].

Therefore, many patients develop protein-energy wasting (PEW), a metabolic syndrome characterized by the loss of body protein and energy stores. PEW is associated with impaired immune function, increased susceptibility to infections, delayed recovery, and higher mortality [7]. Nutritional support therefore represents a fundamental component of the supportive management of patients with AKI, particularly in those undergoing renal replacement therapies, which further increase amino acid and protein losses [8]. Current nutritional recommendations in AKI primarily focus on the quantity of protein intake required to compensate for catabolic losses and extracorporeal removal during dialysis [9,10].

However, increasing evidence suggests that the quality and metabolic characteristics of dietary proteins may also influence renal physiology, inflammation, and metabolic stress. In this context, plant-based proteins have recently gained attention as a potentially advantageous nutritional strategy in kidney disease [11]. Clinical studies, primarily in chronic kidney disease populations, have shown that plant-based dietary patterns are associated with improved metabolic profiles, reduced inflammation, and slower progression of renal dysfunction. Observational data have also suggested a lower risk of mortality and improved renal outcomes among individuals adhering to plant-based diets. Although direct evidence in acute kidney injury remains limited, these findings support the potential role of plant-based proteins as a beneficial nutritional strategy in this setting [12,13,14]. This reflects a growing trend toward functional foods, defined as foods that may provide health benefits beyond basic nutrition, which are increasingly recognized for their potential role in disease prevention and metabolic health [15]. Plant-based protein products are derived from a variety of sources, including soy, pea, rice, wheat, and other legumes and cereals. These sources differ in amino acid composition, digestibility, and bioavailability, which may influence their metabolic effects and clinical implications.

Compared with animal-derived proteins, plant-based proteins are associated with a lower dietary acid load, reduced production of gut-derived uremic toxins, and beneficial modulation of the intestinal microbiota. Moreover, plant-based proteins differ in their amino acid composition, which may influence oxidative stress pathways, endothelial function, and inflammatory responses. These characteristics suggest that plant-derived proteins may contribute to a more favorable metabolic environment for renal recovery [11].

This review examines the role of protein metabolism and nutritional support in AKI, with a particular focus on the potential nephroprotective effects of plant-based proteins and their implications for clinical nutrition in critically ill patients.

## 2. Pathophysiology and Nutritional Balance in AKI

**Figure 1:**
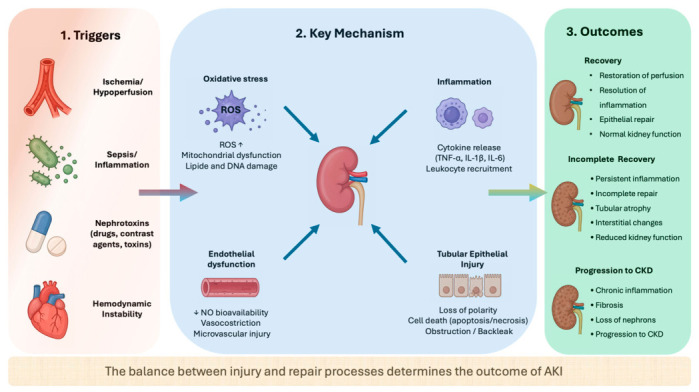
**Pathophysiology of acute kidney injury: key mechanisms and outcomes.** Acute kidney injury (AKI) may result from multiple triggers, including ischemia/hypoperfusion, sepsis, nephrotoxicity, and hemodynamic instability. These insults activate key biological pathways such as oxidative stress, inflammation, endothelial dysfunction, and tubular epithelial injury. Oxidative stress is characterized by increased reactive oxygen species (ROS) production and mitochondrial dysfunction, while inflammation involves cytokine release and leukocyte recruitment. Endothelial dysfunction contributes to microvascular injury and impaired perfusion, and tubular epithelial injury is associated with loss of polarity, cell death, and tubular obstruction. The balance between injury and repair processes ultimately determines clinical outcomes, ranging from complete recovery to incomplete repair and progression to chronic kidney disease (CKD).

In acute kidney injury (AKI), maladaptive repair processes may promote progression to chronic kidney disease (CKD). These include tubular epithelial injury, endothelial dysfunction, and interstitial inflammation leading to fibrosis [16,17,18]. Renal hypoxia, due to the kidney’s high energy demands especially in proximal tubular cells, exacerbates these changes [3,17]. Tubular cells adapt by shifting metabolism from fatty acid oxidation to glycolysis to survive hypoxic stress, yet persistent metabolic alterations promote inflammation, lipid accumulation, and fibrosis, accelerating CKD progression [17]. These mechanisms are summarized in Figure 1.

Inflammation and oxidative stress play a fundamental role in the pathogenesis of this condition. Injured renal epithelial cells release proinflammatory cytokines such as TNF-α and IL-6 that recruit leukocytes, amplifying renal inflammation [18,19].

Mitochondrial dysfunction further impairs energy metabolism, driving the AKI-to-CKD transition [20]. Mitochondria produce reactive oxygen species (ROS) during AKI; excess mitochondrial ROS damage mitochondrial components, impairing bioenergetics and inducing cell death [21,22]. This oxidative stress fuels persistent inflammation and defective repair processes, playing a pivotal role in both acute injury and progression to CKD. These pathophysiological alterations have direct implications for nutritional management in AKI patients.

Concurrently, AKI disrupts the renal regulation of fluid, electrolytes, and acid-base balance, complicating nutritional care. Fluid management must be carefully individualized to avoid hypovolemia or fluid overload, which can worsen renal function and outcomes [23]. Electrolyte imbalances, like hyperkalemia, or metabolic acidosis are common and require vigilant monitoring and correction. Nutritional support should consider fluid status, with restrictions during oliguric phases and modifications during recovery phases characterized by polyuria. Micronutrient levels also require careful oversight to optimize patient management during the dynamic phases of AKI [8,23].

Metabolically, critically ill patients with AKI enter a catabolic state characterized by increased protein breakdown and decreased synthesis, leading to muscle wasting and negative nitrogen balance [4,5]. Amino acids released from muscle are redirected to vital organs such as the liver and gut for gluconeogenesis and acute phase protein synthesis to support immune function. This protein catabolism correlates with illness severity and is linked to mitochondrial dysfunction and inflammatory signaling in skeletal muscle [4]. Therefore, protein provision in critically ill patients with AKI must be carefully balanced to support recovery while limiting further catabolism. This requires considering not only the quantity but also the quality of protein intake, which plays a critical role in modulating renal damage and outcomes. Plant-based proteins generally exert protective effects compared to animal proteins, particularly red and processed meats: plant-based diets may improve kidney function and reduce inflammation due to their content of beneficial minerals and antioxidants [14,24]. Clinical evidence, mainly derived from observational studies in CKD populations (including cohorts ranging from several hundred to thousands of patients), suggests that plant-based diets are associated with slower CKD progression and reduced mortality risk [25,26]. However, data specifically addressing patients with AKI remain limited, and most available evidence is extrapolated from CKD or general population studies. While animal proteins have a higher biological value, diverse sources of plant-based proteins can adequately meet nutritional needs without adverse effects [26]. Furthermore, plant-based diets are associated with lower albuminuria and better cardiovascular outcomes, reinforcing their role in renal nutritional strategies [26]. Overall, integrating metabolic, inflammatory, and hemodynamic considerations into nutritional strategies is essential to mitigate kidney injury and prevent the progression of AKI-to-CKD transition. A key limitation of the current evidence is the lack of dedicated clinical trials specifically evaluating plant-based protein strategies in patients with AKI, as most available data are derived from CKD or general population studies. In addition, heterogeneity in study design and challenges in standardizing protein intake further limit the generalizability of current findings.

## 3. Gut Microbiota and Kidney Crosstalk in AKI

Growing evidence highlights the bidirectional interaction between the gut microbiota and kidney function, often referred to as the gut–kidney axis [27]. In the context of acute kidney injury (AKI), alterations in gut microbiota composition (dysbiosis) may contribute to systemic inflammation, metabolic disturbances, and the progression of kidney damage. AKI is associated with increased intestinal permeability, which facilitates the translocation of bacterial products and endotoxins into the circulation, thereby amplifying inflammatory responses and organ dysfunction.

Gut microbiota-derived metabolites play a crucial role in this process. Protein fermentation by proteolytic bacteria leads to the production of uremic toxins such as indoxyl sulfate, p-cresyl sulfate, and trimethylamine N-oxide (TMAO), which have been implicated in oxidative stress, endothelial dysfunction, and renal fibrosis [28]. Conversely, saccharolytic fermentation of dietary fiber promotes the production of short-chain fatty acids (SCFAs), including acetate, propionate, and butyrate, which exert anti-inflammatory and immunomodulatory effects and may contribute to renal protection [29,30].

Although most evidence derives from CKD models and experimental studies, emerging data suggest that modulation of the gut microbiota may represent a promising therapeutic target in AKI. Dietary strategies, particularly plant-based diets rich in fiber, may favorably influence gut microbiota composition and metabolic profiles, potentially mitigating kidney injury and supporting recovery. However, clinical evidence specifically in AKI populations remains limited and warrants further investigation.

## 4. Protein Requirements and Clinical Challenges in AKI

In critically ill patients, acute kidney injury (AKI) is commonly associated with a negative nitrogen balance, resulting from an imbalance between protein synthesis and degradation. This condition is driven by stress-related hypercatabolism induced by systemic inflammation, sepsis, trauma, and acute respiratory distress syndrome (ARDS). Hormonal and cytokine-mediated pathways, including interleukin-1 and interleukin-6 signaling, stimulate skeletal muscle proteolysis, promoting the release of amino acids that are redirected to the liver for gluconeogenesis and acute-phase protein synthesis [4,5,31]. The clinical consequences of persistent protein catabolism include the loss of lean body mass, impaired immune function, increased susceptibility to infections, and higher mortality risk [31,32]. In patients with AKI, these alterations are further exacerbated by metabolic acidosis, inflammation, and reduced renal function, all of which contribute to increased protein breakdown and impaired anabolic response [4,5].

In patients undergoing continuous renal replacement therapy (CRRT), protein and amino acid losses represent an additional determinant of negative nitrogen balance. Protein losses vary depending on the modality of RRT and membrane characteristics. Convective techniques such as continuous venovenous hemofiltration (CVVH) are associated with greater losses compared with diffusive techniques such as continuous venovenous hemodialysis (CVVHD), while hemodiafiltration results in intermediate losses [33,34]. Overall, protein losses during CRRT are estimated to range from 10 to 25 g/day, with amino acid losses averaging 10–15 g/day and occasionally exceeding 30 g/day in highly catabolic patients [33,35]. Although albumin loss is generally limited, it may become clinically relevant in patients with increased membrane permeability or baseline hypoalbuminemia [36]. These extracorporeal losses further aggravate protein-energy wasting and make achieving a positive nitrogen balance particularly challenging. Current international guidelines emphasize the need to tailor protein intake according to the clinical phase of AKI and the use of RRT. Protein requirements may vary substantially depending on AKI severity and the use of RRT, with higher needs in patients undergoing CRRT compared with non-dialyzed patients.

KDIGO guidelines recommend a protein intake of approximately 0.8 g/kg/day in patients with chronic kidney disease (CKD) stages G3–G5 not requiring dialysis, avoiding excessive protein intake (>1.3 g/kg/day) to limit disease progression. However, in the presence of AKI and especially during RRT, protein requirements are substantially increased to compensate for catabolic losses.

Similarly, ESPEN and ERN-ERA guidelines recommend a protein intake of 0.8–1.0 g/kg/day in stable CKD patients, while advising against protein restriction in the presence of protein-energy wasting. In critically ill patients with AKI undergoing CRRT, protein provision should be increased to 1.5–1.8 g/kg/day, and up to 2.5 g/kg/day in cases of severe hypercatabolism, in order to achieve or approach a positive nitrogen balance [7,34].

Despite the increased protein requirements associated with hypercatabolism and extracorporeal losses, emerging evidence suggests that very high protein intake during the early phase of critical illness may not improve outcomes and may even be harmful.

Randomized clinical trials such as EFFORT-Protein [37], PRECISE [38], and EAT-ICU [39] have shown that early high protein delivery (≥2.0–2.2 g/kg/day) does not confer functional or survival benefits compared with moderate protein intake and may be associated with worse long-term quality of life. In particular, early aggressive protein administration during the first 48–72 h of critical illness does not improve recovery and may increase metabolic burden [37,38,39]. These findings support a phase-adapted nutritional strategy, in which protein intake is progressively increased over time. A moderate initial intake (approximately 0.8–1.2 g/kg/day) during the acute phase, followed by higher protein provision during the recovery phase, appears to be the most physiologically appropriate approach [40].

## 5. Plant-Based Proteins in Enteral Nutrition: Clinical Potential

Enteral nutrition (EN) is the preferred route for nutritional support in critically ill patients, as it preserves gut integrity, modulates immune function, and is associated with lower infectious complications compared with parenteral nutrition. Current guidelines recommend early initiation of EN whenever the gastrointestinal tract is functional, making the composition of enteral formulas a key determinant of clinical outcomes. In this context, plant-based enteral formulas have gained increasing attention. For example, COMPLEAT^®^ Plant Protein (Nestlé Health Science) is a commercially available enteral formula based on plant-derived proteins, fibers, and whole-food ingredients. Similar plant-based enteral formulations are also available, although their composition and clinical evidence may vary. It provides a balanced macronutrient profile including proteins from pea and soy, complex carbohydrates, and a blend of soluble and insoluble fibers, and is suitable for patients with lactose intolerance or those requiring plant-based nutritional strategies.

From a gastrointestinal perspective, plant-dominant formulas may offer improved tolerance compared with casein-based formulations. In vitro and clinical data suggest that plant-based proteins do not coagulate in the gastric environment, potentially facilitating gastric emptying and reducing feeding intolerance in critically ill patients [41]. In addition, the fiber content of plant-based formulas supports gut motility, enhances microbiota diversity, and reduces the incidence of diarrhea and constipation frequently observed in ICU patients receiving enteral nutrition.

From a metabolic and renal perspective, plant-derived proteins are associated with a lower dietary acid load, improved nitrogen handling, and reduced renal hemodynamic stress. In patients with kidney disease, adherence to plant-based dietary patterns has been associated with an improved estimated glomerular filtration rate and a significantly lower risk of CKD [13], as well as favorable metabolic and inflammatory profiles [13]. Moreover, plant-based diets have been linked to reduced mortality and improved cardiometabolic outcomes in CKD populations [12]. However, it should be acknowledged that much of the available evidence derives from studies in chronic kidney disease (CKD) or general populations, and direct evidence in AKI remains limited. Overall, plant-based enteral nutrition represents a promising strategy to combine gastrointestinal tolerance, metabolic optimization, and renal protection in critically ill patients. Further randomized studies are needed to evaluate their impact on clinical outcomes such as infection rates, muscle preservation, and long-term recovery.

## 6. Amino Acid Composition: Comparison of Plant-Based Proteins

The nutritional quality of dietary proteins is primarily determined by their content of essential amino acids in relation to human requirements, as defined by the WHO/FAO/UNU reference pattern. Compared with animal-derived proteins, individual plant-based proteins may present deficiencies in one or more essential amino acids, most commonly lysine, methionine, or leucine. However, these limitations vary substantially among plant sources and can be overcome through appropriate combinations of different protein sources [42].

Among plant-based proteins, soy protein is generally considered the highest-quality source, as its amino acid profile closely approaches WHO/FAO standards and achieves high protein quality scores (Protein Digestibility Corrected Amino Acid Score (PDCAAS) close to 1.0). Pea protein is characterized by a high lysine content but relatively fewer sulfur-containing amino acids (methionine and cysteine), whereas rice protein is typically limited in lysine but relatively rich in methionine. These complementary patterns explain why individual plant-based proteins may appear “incomplete” when assessed in isolation [43].

Importantly, combining legume proteins (such as soy or pea, rich in lysine) with cereal proteins (such as rice, richer in methionine) allows the formulation of **complete amino acid profiles** that meet World Health Organization (WHO) and Food and Agriculture Organization (FAO) requirements. Evidence shows that blends of plant-based proteins can achieve protein quality values comparable to animal-derived proteins, including PDCAAS values close to 1.0 when appropriately balanced [44].

From a metabolic standpoint, amino acid composition also influences the anabolic response. Plant-based proteins often contain lower leucine concentrations compared with whey protein, potentially attenuating muscle protein synthesis when consumed as a single source. However, this limitation can be mitigated by combining different plant-based proteins or by leucine enrichment, restoring an anabolic response comparable to that of animal proteins [42].

The comparative amino acid profile of soy, pea, and rice proteins relative to WHO/FAO reference values is reported in Table 1.

Each plant protein source presents a distinct amino acid profile with specific strengths and limiting components. Soy protein shows the most balanced composition, with essential amino acids generally meeting WHO/FAO requirements. Pea protein is particularly rich in lysine but relatively limited in sulfur-containing amino acids (methionine and cysteine), whereas rice protein contains higher levels of methionine but lower lysine content. These complementary characteristics explain why combining legume and cereal proteins allows the achievement of a balanced essential amino acid profile capable of meeting human requirements [45].

These complementary amino acid patterns highlight an important nutritional principle that meets human requirements. From a clinical perspective, this aspect is particularly relevant in critically ill patients with AKI, in whom adequate amino acid availability is essential to sustain protein synthesis, preserve lean body mass, and support immune and metabolic functions. Accordingly, plant-based enteral formulas that combine multiple protein sources can provide adequate amino acid quality while potentially offering additional metabolic advantages, including a lower dietary acid load and reduced renal hemodynamic stress [23,46].

## 7. Nephrotoxic and Protective Amino Acids

Amino acids play a central role in renal physiology, as the kidney actively participates in their filtration, tubular reabsorption, and metabolic processing [47]. Under physiological conditions, almost all filtered amino acids are efficiently reabsorbed in the proximal tubule through specific transport systems, preventing urinary loss and contributing to systemic nitrogen balance [48]. In kidney disease, however, both glomerular and tubular dysfunction may alter these processes, leading to disturbances in amino acid metabolism, aminoaciduria, and the accumulation of potentially harmful metabolites [48].

The potential nephrotoxic effects of certain amino acids are not related to their tubular reabsorption per se, but rather to alterations in their metabolic pathways and the generation of pro-oxidative or uremic metabolites. In particular, amino acids such as methionine, phenylalanine, and tyrosine have been associated with increased oxidative stress, the formation of advanced glycation end products (AGEs), and the activation of inflammatory and fibrotic signaling pathways, which may contribute to endothelial dysfunction and renal injury [47]. Experimental evidence supports this concept: in a model of ischemia–reperfusion kidney injury, dietary methionine restriction reduced macrophage infiltration and attenuated tubular injury and interstitial fibrosis, suggesting a role for methionine metabolism in renal immunometabolic responses [49].

Conversely, several amino acids appear to exert protective effects on renal and endothelial function. Glycine, alanine, and serine have antioxidant and cytoprotective properties, while arginine serves as a precursor for nitric oxide synthesis, supporting endothelial function and renal microcirculation. Taurine has also been associated with anti-inflammatory and membrane-stabilizing effects. In addition, the dysregulation of amino acid metabolic pathways has been linked to increased oxidative stress and ferroptotic cell death in renal tissue, further highlighting the role of amino acid metabolism in kidney injury mechanisms [50]. These qualitative differences in amino acid function and metabolism are summarized in Table 2, which distinguishes amino acids potentially associated with pro-oxidative pathways from those with cytoprotective or anti-inflammatory effects.

## 8. Recent Evidence and Clinical Implications

Beyond their amino acid composition, the physiological effects of different protein sources may also influence renal hemodynamics, metabolic pathways, and inflammatory responses. Clinical studies have demonstrated an association between high animal protein intake and the development of renal hyperfiltration (RHF), a condition that appears to represent one of the mechanisms of CKD progression and has been suggested as a marker of all-cause mortality in a healthy general population [53,54].

Conversely, in individuals at risk of developing AKI, amino acid administration may exert protective effects. This occurs through an increase in renal blood flow and enhanced tubular sodium reabsorption, which reduces signals for tubuloglomerular feedback activation. The resulting decrease in afferent arteriolar resistance may transiently protect the kidney from acute damage caused by hypoperfusion or hypoxia [55].

However, once AKI has occurred, persistent hyperfiltration may contribute to the progression toward chronic kidney injury [56]. Dietary acid load has also been associated with the development of RHF in the general population [57]. Plant-based diets (PBDs), which are naturally low in or exclude animal products, consistently reduce dietary acid load and may therefore exert nephroprotective effects [52]. Compared with animal protein sources, plant-derived proteins contain phosphate that is less efficiently absorbed and are naturally rich in dietary fiber [51,58]. These characteristics may be beneficial in AKI. During AKI, both serum phosphate and fibroblast growth factor-23 (FGF23) increase rapidly and are independently associated with disease severity and outcomes. Experimental evidence suggests that dietary phosphate restriction may reduce calciprotein particle formation, inflammation, acidosis, cardiac electrical disturbances, and mortality in murine models of AKI [59,60].

Increasing dietary fiber intake has also been associated with reduced levels of uremic toxins and inflammatory mediators, as well as improved intestinal barrier function in CKD patients. A recent meta-analysis showed that higher fiber intake is associated with reduced all-cause mortality, cardiovascular mortality, and cardiovascular disease risk in CKD populations [61]. In addition, short-chain fatty acids (SCFAs) produced by the microbial fermentation of dietary fiber exert anti-inflammatory and immunomodulatory effects that may protect against AKI [29]. In murine models, supplementation with Lactobacillus casei reduced the severity of kidney injury by increasing SCFA and nicotinamide production [27,30].

**Figure 2:**
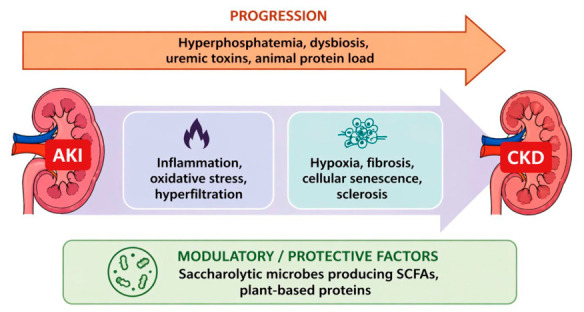
**Simplified schematic representation of the pathophysiological mechanisms linking acute kidney injury (AKI) to chronic kidney disease (CKD) progression and the potential modulatory role of plant-based dietary factors.** AKI may progress to CKD under the influence of metabolic and environmental factors, including hyperphosphatemia, gut dysbiosis, uremic toxin accumulation, and high animal protein intake. These factors promote key pathological processes such as inflammation, oxidative stress, hyperfiltration, hypoxia, fibrosis, cellular senescence, and sclerosis. In parallel, plant-based dietary patterns may exert protective effects by promoting saccharolytic gut microbiota and short-chain fatty acid (SCFA) production, thereby modulating inflammation and metabolic stress. The balance between injurious and protective mechanisms may influence renal recovery or progression to CKD.

Plant-based proteins also contain lower amounts of carnitine, choline, phosphatidylcholine, tyrosine, and tryptophan—precursors of gut-derived uremic toxins such as trimethylamine N-oxide (TMAO), p-cresyl sulfate (PCS), and indoxyl sulfate (IS), which are associated with cardiovascular and renal toxicity [58]. The mechanisms linking animal protein load, gut-derived uremic toxins, and kidney injury progression are illustrated in Figure 2.

In AKI, TMAO has been shown to promote renal inflammation, apoptosis, oxidative stress, and functional impairment [62]. Similarly, indoxyl sulfate contributes to AKI-to-CKD progression through mechanisms involving TGF-β signaling activation, oxidative stress, inflammation, cellular senescence, and suppression of Klotho expression through epigenetic regulation [28].

Finally, experimental studies suggest that caloric restriction, often associated with predominantly plant-based dietary patterns in humans, may also exert protective effects against AKI [63,64,65].

## 9. Future Perspectives

Despite increasing interest in the role of nutrition in acute kidney injury (AKI), evidence specifically addressing the impact of different protein sources on renal outcomes remains limited. Most current nutritional recommendations focus primarily on protein quantity, while the potential influence of protein quality and amino acid composition on renal physiology, inflammation, and metabolic stress has received comparatively less attention. In particular, the role of plant-based proteins in modulating renal hemodynamics, oxidative stress, and gut–kidney axis interactions represent an emerging area of research.

Future studies should aim to clarify whether plant-derived protein sources may confer clinically relevant nephroprotective effects in critically ill patients with AKI. Randomized controlled trials comparing plant-based and animal-derived protein formulations in enteral nutrition could help determine their impact on important clinical outcomes, including renal recovery, need for RRT, preservation of lean body mass, and long-term kidney function. In addition, the interaction between dietary proteins and the intestinal microbiota deserves further investigation, as plant-based diets may influence the production of gut-derived uremic toxins and short-chain fatty acids, both of which have been implicated in kidney injury and recovery.

**Figure 3:**
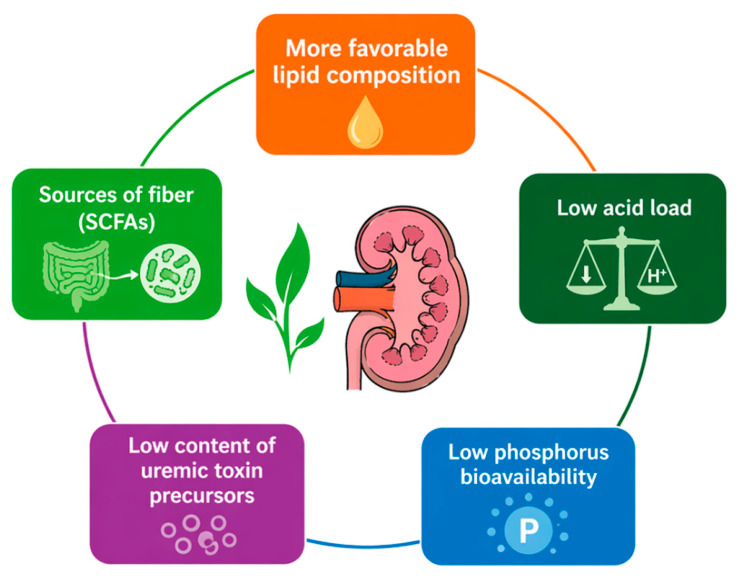
**Simplified schematic representation of the potential metabolic mechanisms through which plant-based dietary patterns may influence kidney function and disease progression.** Plant-based protein sources may exert nephroprotective effects through several proposed metabolic pathways. Compared with animal-derived proteins, plant-based proteins are generally characterized by a lower content of uremic toxin precursors, reduced phosphorus bioavailability, and higher dietary fiber content. Increased fiber intake may promote the growth of saccharolytic gut microbiota and the production of short-chain fatty acids (SCFAs), which are associated with anti-inflammatory and immunomodulatory effects. In addition, plant-based dietary patterns are typically associated with a lower dietary acid load and a more favorable lipid profile. Overall, these mechanisms may contribute to a more favorable metabolic environment; however, most of these effects are inferred from CKD or experimental studies rather than direct evidence.

Advances in metabolomics and nutritional phenotyping may also allow a more precise characterization of amino acid metabolism during AKI, potentially enabling personalized nutritional strategies tailored to the metabolic state of critically ill patients. Ultimately, integrating nutritional interventions with a deeper understanding of renal metabolism and the gut–kidney axis may open new therapeutic perspectives aimed at improving outcomes and preventing the transition from AKI to chronic kidney disease. The potential metabolic pathways through which plant-based dietary patterns may exert nephroprotective effects are summarized in Figure 3.

## 10. AKI Limitations

This review has several limitations. Most of the available evidence regarding the effects of plant-based proteins on renal outcomes is derived from studies in chronic kidney disease or general populations, and direct evidence in AKI remains limited. In addition, heterogeneity in patient populations, disease severity, and the use of renal replacement therapy makes it challenging to standardize protein intake and nutritional strategies. Further AKI-specific clinical trials are needed to validate these findings and to define optimal nutritional approaches in this setting.

## 11. Conclusions

Acute kidney injury (AKI) in critically ill patients is characterized by profound metabolic alterations, including hypercatabolism, negative nitrogen balance, and protein-energy wasting. Nutritional support therefore represents a crucial component of supportive therapy, particularly in patients undergoing RRT. While current recommendations mainly focus on the amount of protein intake required to compensate for catabolic losses, growing evidence suggests that the quality and metabolic characteristics of dietary protein sources may also influence renal physiology and recovery. Plant-based proteins represent a promising nutritional strategy in this context. Compared with animal-derived proteins, plant-based proteins are associated with a lower dietary acid load, reduced production of gut-derived uremic toxins, and beneficial modulation of the gut microbiota. Their amino acid composition may also influence oxidative stress, inflammatory pathways, and renal hemodynamics. Together, these mechanisms may contribute to a more favorable metabolic environment and potentially support renal recovery. Future clinical studies are needed to determine whether plant-based protein formulations in enteral nutrition can improve clinical outcomes and reduce the risk of AKI-to-CKD transition in critically ill patients.

## Figures and Tables

**Table 1:** Essential amino acid composition of soy, pea, and rice proteins compared with the WHO/FAO reference pattern.

Essential Amino Acid (g/100 g Protein)	Soy Protein	Pea Protein	Rice Protein	WHO/FAO Reference Pattern *
Leucine	7.6	6.8	6.9	6.6
Lysine	6.4	7.2	3.7	5.8
Valine	5.2	5.0	5.2	5.0
Methionine + Cysteine	2.4	2.2	3.8	2.5
Phenylalanine + Tyrosine	8.3	6.4	7.0	6.3
Threonine	3.9	4.1	3.2	3.4
Tryptophan	1.2	0.9	1.1	0.9
Isoleucine	4.2	4.5	3.9	3.0
Histidine	2.6	2.5	2.3	1.9

Notes: * WHO/FAO/UNU reference pattern for essential amino acid requirements in adults (expressed as mg/g protein equivalent and converted here to g/100 g protein). Values for plant-based proteins derived from USDA FoodData Central and published compositional analyses of soy, pea, and rice protein isolates. Notes: Data adapted from published guidelines and clinical recommendations [9,10,34].

**Table 2:** Amino acids potentially associated with pro-oxidative or protective effects in kidney injury.

Category	Amino Acids	Main Biological Mechanisms	Potential Renal Effects
**Pro-oxidative/potentially nephrotoxic**	Methionine	Sulfur amino acid metabolism; increased methylation pathways and oxidative stress	AGE formation, ROS production, inflammatory signaling
	Phenylalanine	Aromatic amino acid metabolism; oxidative pathways	Endothelial dysfunction, oxidative stress
	Tyrosine	Catecholamine and oxidative metabolism	Cellular stress and inflammatory activation
	Tryptophan	Generation of uremic toxins (e.g., indoxyl sulfate, kynurenine metabolites)	Tubular injury, oxidative stress, fibrosis
**Protective/cytoprotective**	Glycine	Anti-inflammatory signaling; glutathione synthesis	Reduced oxidative stress, tubular protection
	Alanine	Metabolic buffering and gluconeogenesis support	Improved cellular metabolic stability
	Serine	Precursor for antioxidant pathways and one-carbon metabolism	Cytoprotection and redox balance
	Arginine	Nitric oxide precursor	Improved endothelial function and renal microcirculation
	Taurine	Membrane stabilization; antioxidant activity	Reduced inflammation and oxidative injury

**Notes:** AGE = advanced glycation end products; ROS = reactive oxygen species. Classification based on experimental and mechanistic evidence linking amino acid metabolism with oxidative stress, inflammation, and renal injury. Data adapted from studies on plant-based proteins and kidney disease [11,14,51,52].

## Data Availability

No new data were created or analyzed in this study. Data sharing is not applicable to this article.

## Abbreviations

The following abbreviations are used in this manuscript:

AKI	Acute kidney injury
ARDS	Acute respiratory distress syndrome
CKD	Chronic kidney disease
CRRT	Continuous renal replacement therapy
CVVHD	Continuous venovenous hemodialysis
CVVH	Continuous venovenous hemofiltration
EN	Enteral nutrition
FAO	Food and Agriculture Organization
FGF23	Fibroblast growth factor 23
ICU	Intensive care unit
IS	Indoxyl sulfate
PCS	p-cresyl sulfate
PDCAAS	Protein Digestibility Corrected Amino Acid Score
PEW	Protein-energy wasting
RRT	Renal replacement therapy
ROS	Reactive oxygen species
SCFAs	Short-chain fatty acids
TMAO	Trimethylamine N-oxide
UNU	United Nations University
WHO	World Health Organization
